# The DosS-DosT/DosR Mycobacterial Sensor System

**DOI:** 10.3390/bios3030259

**Published:** 2013-07-04

**Authors:** Santhosh Sivaramakrishnan, Paul R. Ortiz de Montellano

**Affiliations:** Department of Pharmaceutical Chemistry, School of Pharmacy, University of California, San Francisco, CA 94158, USA; E-Mail: santhosh.sivaramakrishnan@ucsf.edu

**Keywords:** DevS, DosS, DosT, DosR, *Mycobacterium tuberculosis*, heme-based sensors, two-component system, nitric oxide, carbon monoxide, hypoxia, dormancy

## Abstract

DosS/DosR is a two-component regulatory system in which DosS, a heme-containing sensor also known as DevS, under certain conditions undergoes autophosphorylation and then transfers the phosphate to DosR, a DNA-binding protein that controls the entry of *Mycobacterium tuberculosis* and other mycobacteria into a latent, dormant state. DosT, a second sensor closely related to DosS, is present in *M. tuberculosis* and participates in the control of the dormancy response mediated by DosR. The binding of phosphorylated DosR to DNA initiates the expression of approximately fifty dormancy-linked genes. DosT is accepted to be a gas sensor that is activated in the ferrous state by the absence of an oxygen ligand or by the binding of NO or CO. DosS functions in a similar fashion as a gas sensor, but contradictory evidence has led to the suggestion that it also functions as a redox state sensor. This review focuses on the structure, biophysical properties, and function of the DosS/DosT heme sensors.

## 1. Introduction

*Mycobacterium tuberculosis* (*Mtb*) can survive *in vivo* for prolonged periods of time (decades) in a clinically undetectable latent state called non-replicating persistence. *Mtb* is metabolically dormant in the latent state and is unresponsive to most antituberculosis drugs. Diverse stimuli, including hypoxia [1], exposure to nitric oxide [2], nutrient deprivation [3], and increased acidity of the microenvironment [4] are thought to trigger dormancy, which can be reversed when the host immune response is compromised, as in HIV co-infection [5].

Early evidence linked hypoxia to dormancy. Wayne established that tuberculosis bacteria settle into a non-replicating, persistent state when grown under low oxygen tension or hypoxia and, more importantly, that the bacilli developed resistance to clinical drugs [1]. Tuberculosis infections are normally localized in the oxygen rich regions of the lungs, which indicates that normal *in vivo* growth and survival requires oxygen. However, mycobacteria also experience hypoxic conditions *in vivo* inside macrophages. Thus, *Mtb* growth is inhibited when the bacilli are engulfed into granulomas, the inside of which is associated with low oxygen levels [6]. Similarly, oxygen depletion has been shown to inhibit the growth of *Mycobacteria bovis* BCG and to result in dormancy [7].

Genetic analysis combined with studies of *Mtb* cultures grown *in vitro* under limited oxygen conditions demonstrated the enhanced expression of a 16 KDa α-crystallin homolog protein (Acr) [8]. Acr is a small heat shock protein with a chaperonin activity that is selectively expressed under hypoxic conditions and is required for the growth of *Mtb* in macrophages [9]. This protein has been used as a marker to identify the dormancy state of the bacilli. While expression of the Acr protein under low oxygen conditions was evident, the genes that are responsible for the continued growth of the bacilli in stationary cultures under hypoxia were not known at the time.

In independent work, the gene products encoding a two-component system with a cognate response regulator were identified using extensive genetic analysis combined with subtractive hybridization [1,10]. Importantly, this system was expressed at higher levels in the virulent H37Rv strain compared to the avirulent H37Ra strain [11,12]. Thus, the differentially expressed gene products in virulent strain were called *dev* genes and the region representing the two-component system with the response regulator was named *devR*-*devS* [10]. The expressed proteins led to the identification of gene products Rv3132c and Rv3133c that encode, respectively, a 578 amino acid histidine kinase protein (termed DevS) and a 217 amino acid response regulator protein (termed DevR). The gene product downstream of the *devR*-*devS* loci, Rv3134c, encodes a well-conserved Ala-Val rich protein whose function remains unknown. However RT-PCR analysis of the RNA isolated from the *Mtb* cultures using the primers for the *devR*-*devS* locus suggests that the genes in the region are co-transcribed and that *devS*-*devR*-*Rv3134c* form an operon [11].

In order to establish the hypoxia-specific expression of the Acr protein, Sherman and coworkers utilized whole-genome microarray analyses to examine the array of genes expressed when *Mtb* is exposed to reduced oxygen tension [13]. The Acr protein was upregulated under hypoxia along with a set of 47 other genes (Table 1). It is now evident that the DevS/DevR two-component system is responsible for the induction of approximately 48 genes in response to conditions such as hypoxia or exposure to nitric oxide or carbon monoxide [2,14,15]. Induction of this panel of genes is required for *Mtb* survival under these conditions [13,16]. The predicted function of most of the genes suggests a role for them in adaptation of* Mtb* to a lower metabolic activity under hypoxia. A similar hypoxic induction of *devR*-*devS* has been observed for BCG grown in an *in vitro* dormancy model [17]. Importantly, disruption of the *Rv3133c* gene failed to regulate the expression of other proteins responsible for its survival under hypoxia, indicating that the DevR protein is essential for dormancy survival and regulation in BCG. Thus DevR was given the alternative name DosR that stands for DOrmancy Survival Regulator protein and the system the name DosS/DosR [18]. In the rest of this review, we will exclusively utilize the DosS/DosR nomenclature. Furthermore, knockout of both *dosS* and *dosT* in *Mtb* yields bacteria that cannot upregulate the expression of *dosR* activated genes [13]. Studies of the *Mtb dosR* knockout show that it is not essential *in vitro* for entry, survival, or multiplication of *Mtb* in human monocytes [19], although it has been shown to contribute to virulence in guinea pigs. The role of the *dosR* system in virulence appears to be model dependent [20,21,22], but in general contributes to survival and growth under hypoxic conditions.

Comparison of the genome sequences of *Mtb* and *M. smegmatis* reveals the presence in the latter of genes that correspond to *dosS* and *dosR* [23]. The two genes are upregulated during hypoxia, suggesting that the system for response to hypoxia is similar in both *Mtb* and *M. smegmatis*. However, *M. smegmatis* does not have a gene that corresponds to *dosT* [24,25], although phylogenetically the *M. smegmatis* *dosS* is more closely related to *Mtb* *dosT* than *dosS* [24]. 

DosS and DosT are sensor kinases that autophosphorylate one of their own histidines and subsequently use the phosphorylated histidine to phosphorylate an aspartate residue of DosR, resulting in binding of DosR to DNA upstream of hypoxic response genes and thus activating the *dosR* regulon [13,26]. DosT encodes a protein that is 62.5% identical to DosS. Its cytosolic C-terminal domain, termed Rv2027c_194_, has been expressed in *E. coli* and shown to undergo autophosphorylation at a conserved histidine (His392) and to transfer the phosphate group to DosR [27]. Unlike DosS, however, DosT can utilize Ca^2+^ in addition to Mg^2+^ as the divalent ion for autophosphorylation. Phosphate transfer from DosT to DosR was slower and went to a lower extent than from DosS, with only partial transfer after 30 min compared to complete transfer by DosS in 5–10 min [27]. These studies established, furthermore, that *dosT* transcription, unlike that of *dosS*, is not upregulated during hypoxia. 

**Figure 1:**
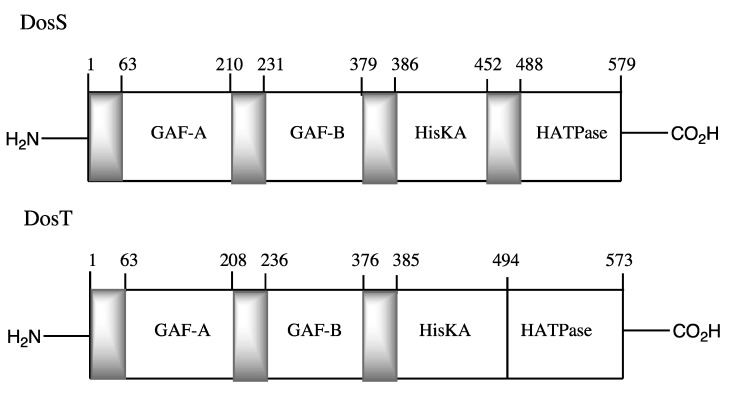
Domain structure of *Mtb* DosS [28] and DosT [27].

The full-length protein structures of DosS and DosT incorporate two N-terminal GAF domains, termed GAF-A and GAF-B, followed by a histidine kinase domain that includes the conserved histidine residue that is phosphorylated, and finally by an ATPase domain (Figure 1). Here, we review the structure and function of the two component DosS-DosT/DosR system, their specific roles in regulating the necessary genes responsible for survival of the *bacilli* under hypoxia, and the contradictory evidence concerning the role of DosS as a gas *versus* redox sensor.

**Table 1:** List of Mtb genes induced under hypoxia [8].

	Gene	↑ Ratio	Gene product
Rv0079		11.6 ± 3.5	HP
Rv0080		7.6 ± 2.1	HP
Rv0081		3.5 ± 1.1	Transcriptional regulator
Rv0569		18.4 ± 4.0	CHP
Rv0572c		13.9 ± 7.8	HP
Rv0574c		3.8 ± 1.7	CHP
Rv1264		2.4 ± 0.2	Similar to adenylate cyclases
Rv1592c		3.1 ± 0.7	CHP
Rv1733c		12.6 ± 4.1	Possible membrane protein
Rv1734c		9.8 ± 7.8	HP
Rv1736c	narX	3.0 ± 0.8	Fused nitrate reductase
Rv1737c	narK2	12.0 ± 3.0	Nitrite extrusion protein
Rv1738		63.3 ± 37	CHP
Rv1739c		3.9 ± 1.1	Possible sulfate transporter
Rv1813c		14.7 ± 9.8	CHP
Rv1997	ctpF	8.8 ± 6.0	Probable cation transport ATPase
Rv1998c		4.7 ± 1.2	CHP
Rv2003c		11.4 ± 7.2	CHP
Rv2005c		8.5 ± 2.7	CHP
Rv2007c	fdxA	22.0 ± 9.9	Ferredoxin
Rv2028c		3.3 ± 1.2	CHP
Rv2029c	pfkB	12.2 ± 6.9	Phosphofructokinase II
Rv2030c		19.1 ± 14	CHP
Rv2031c	acr; hspX	13.6 ± 3.1	14-kDa antigen, heat shock protein
Rv2032		43.9 ± 16	CHP
Rv2428	ahpC	3.8 ± 1.2	Alkyl hydroperoxide reductase
Rv2623		6.8 ± 2.3	CHP
Rv2624c		44.3 ± 34	CHP
Rv2625c		6.3 ± 2.8	CHP
Rv2626c		37.4 ± 7.4	CHP
Rv2627c		17.0 ± 6.3	CHP
Rv2628		4.8 ± 1.1	HP
Rv2629		6.8 ± 1.3	HP
Rv2630		3.9 ± 1.1	HP
Rv2659c		3.7 ± 1.5	PhilRV2 integrase
Rv3126c		20.9 ± 7.3	HP
Rv3127		33.1 ± 14	CHP
Rv3128c		11.7 ± 4.6	CHP
Rv3129		38.6 ± 15	CHP
Rv3130c		26.6 ± 16	CHP
Rv3131		4.3 ± 1.1	CHP
Rv3132c		9.1 ± 3.9	Sensor histidine kinase
Rv3133c		13.8 ± 10	Two-component response regulator
Rv3134c		10.6 ± 2.5	CHP
Rv3841	bfrB	8.1 ± 2.9	Bacterioferritin
Rv3842c	glpQ1	6.9 ± 1.4	Phosphodiesterase
Rv3908		3.7 ± 1.5	CHP

## 2. Heterologous Expression and Heme Binding of DosS and DosT

Early efforts to clone and express the full-length DosS protein were not successful due to difficulties in solubilizing the protein [26]. The *dosS* gene exhibits homology with histidine kinases and sequence analysis initially suggested that DosS might have three transmembrane domains [11], a prediction not borne out by the subsequent expression, purification, and characterization of the protein. The constructs and approaches used to purify the various domain constructs of DosS and DosT are summarized below because discrepancies in the properties of the proteins detailed in later sections of this review may stem from differences in the proteins employed in the different laboratories. 

Dos_201_, the truncated C-terminal histidine kinase domain of DosS consisting of 201 amino acids was first expressed by Saini *et al*. in *E. coli* as a His(6)-tagged protein that formed inclusion bodies [12,29]. The refolded protein was shown to retain both autophosphorylation activity and the ability to transfer the phosphate to DosR [12], establishing that the isolated kinase domain was fully functional. 

In a more extensive study, five poly-His tagged constructs of *Mtb* DosS were cloned by Sardiwal *et al*. [28], including the full-length protein (residues 1–579) and constructs consisting of residues 1–379 (GAF-A + GAF-B), 63–379 (GAF-A + GAF-B), 63–210 (GAF-A), and 231–379 (GAF-B). Each of these constructs was expressed in BL21(DE3) *E. coli* cells as a mixture of soluble and insoluble proteins, from which some soluble protein could be purified. UV-vis spectroscopic analysis of these truncated proteins provided the first evidence that heme is bound to the GAF-A domain [28]. 

Subsequently, the N-terminal GAF-A domain of DosS consisting of residues 63 to 210 plus a six-His tag was expressed in *E. coli* in a pET23a + vector [30]. To improve the expression, the protein was coexpressed at 18 °C together with the GroEL/ES chaperones. The resulting soluble protein was shown by the pyridine hemochrome assays to bind heme with a 1:1 stoichiometry and with a spectroscopically-determined affinity of K_d_ = 3.1 ± 2.0 μM. A similar expression system was used to produce a construct consisting of the GAF-A plus GAF-B domains, as well as full-length DosS [30,31]. Pyridine hemochrome assays again established that the heme is bound in a 1:1 stoichiometry to full-length DosS, which confirmed that heme is only bound in the GAF-A domain. The truncated DosT GAF-A domain, spanning residues 61-208 fused to a six-His tag, was similarly expressed [32].

Sousa *et al*. expressed full-length DosS and DosT, as well as the DosS GAF-A (residues 1–210) and DosT GAF-A (residues 1–208) domains in a pUC-19 vector [33]. The expressed proteins had no His-tag and were purified by conventional chromatography. These authors also used the pyridine hemochromogen assay to independently establish that heme is bound with a 1:1 stoichiometry to the GAF-A domain of both DosS and DosT [33]. 

Steyn and coworkers cloned the full-length *dosS* and *dosT* genes in-frame with either a His-tag or an 11-kDa small ubiquitin related modifier (SUMO) tag in a pET15b vector [34]. The constructs were expressed in Rosetta (DE3) *E. coli* cells and the soluble fractions of the proteins, purified by nickel affinity chromatography, were confirmed by pyridine hemochromogen assays to bind heme with a 1:1 heme:protein stoichiometry [34]. 

Finally, the Kang laboratory in Korea expressed the DosS GAF-A domain from Asp63 to Arg210 with a poly-His tag separated from the desired protein by a TEV protease site. After expression and TEV cleavage, this yielded a purified protein with five additional residues (GAMDP) at the N-terminus [35]. 

In addition to the work with the *Mtb* proteins, full-length *M. smegmatis* DosS has been expressed in *E. coli* and purified in its ferrous state [25]. 

## 3. DosS/DosT GAF-A Heme Domain Structures

### 3.1. Proximal Ligand

**Figure 2:**
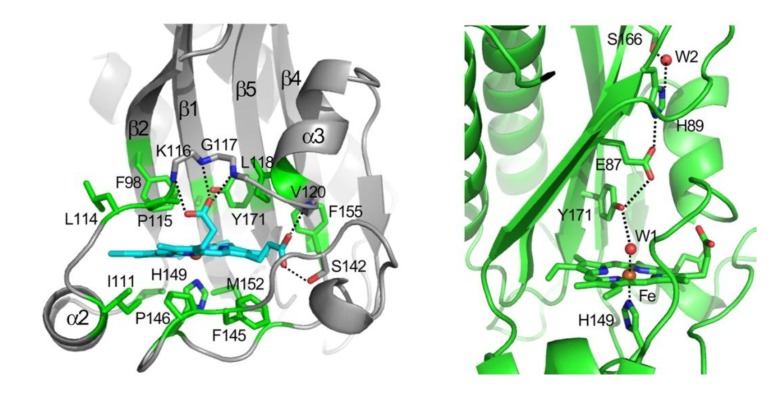
Crystal structure of the ferric DosS GAF-A domain showing the heme surrounded by the hydrophobic residues in a ligand binding pocket. The heme iron is coordinated to His149 to the proximal side and the distal water molecule is hydrogen bonded to the Tyr171 residue, which interacts with the His89 via Glu87 [35]. Reprinted with permission. © 2008 The American Society for Biochemistry and Molecular Biology.

**Figure 3:**
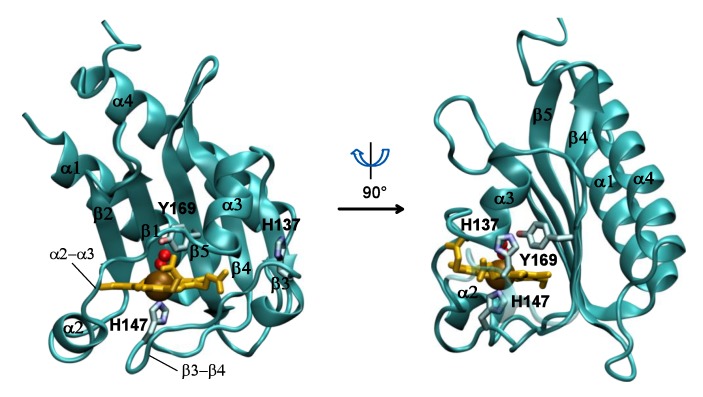
Crystal structure of the ferrous dioxygen complex of the DosT GAF-A domain. Reprinted with permission from [32].

His149 was identified as the proximal iron ligand in *Mtb* DosS by mutating the individual histidines of the truncated GAF-A domain to alanines [28]. Only the H149A mutant did not retain the UV-visible spectrum typical of a His-ligated heme group. A detailed spectroscopic comparison of the H149A mutant with wild-type GAF-A by UV-visible and resonance Raman spectroscopy confirmed the identity of this histidine as the iron ligand [30]. Thus, the ferrous GAF-A domain excited at 442 nm exhibits a resonance Raman scattering at 214 nm characteristic of an Fe-N_His_ bond in which the histidine is neutral and not strongly hydrogen bonded [30]. This inference was supported by the resonance Raman stretching frequencies of the Fe-C and the FeC-O in the complex formed with ^13^CO. The crystal structure of the DosS GAF-A domain showed that the His149-iron Fe-N bond distance was 2.1–2.2 Å (Figure 2) [35]. The crystal structure of the *Mtb* DosT GAF-A domain established that His147, the corresponding histidine residue (Figure 3), is the proximal iron ligand in DosT [32]. Although a crystal structure is not available for the *M. smegmatis* GAF-A domain, site-specific mutagenesis has shown that His150, the equivalent histidine residue, is the iron ligand, as the H150A mutant did not stably bind heme [25]. Allowing for differences in protein length, the same histidine residue is thus the proximal iron ligand in *Mtb* DosS and DosT, as well as *M. smegmatis* DosS. 

### 3.2. Distal Ligand

UV-visible and resonance Raman studies of the truncated GAF-A domain and full-length protein showed that DosS reversibly binds O_2_, CO, and NO. The UV-visible maxima of the GAF-A domains and the full-length proteins with various ligands are summarized in Table 2 [30]. The binding of NO to ferrous full-length DosS and DosT has been confirmed by EPR [34]. The ferric GAF-A domain can also bind cyanide (K_d_ = 0.37 μM) and azide (K_d_ = 8.81 μM), but does not detectably bind large azole agents such as econazole and ketoconazole [30]. Resonance Raman shows that at neutral pH and room temperature, the DosS GAF-A domain exists as a 6-coordinate high-spin complex with a minor component due to a 6-coordinate low-spin complex. Similar UV-visible and resonance Raman spectra were obtained for the GAF-A, GAF-A/GAF-B, and full-length proteins [31]. Upon anaerobic reduction to the ferrous state, the GAF-A domain exists as a 5-coordinate high-spin complex [30]. The resonance Raman modes of the vinyl and propionate groups of the heme in the DosS ferrous-CO complex indicate that these substituents are not perturbed by truncation of the full-length protein [31]. 

**Table 2:** Representative UV-vis maxima for the DosS and DosT constructs from *Mtb* and *M. smegmatis*.

Protein	Fe(III) (nm)	Fe(II) (nm)	Fe(II)-CO (nm)	Fe(II)-NO (nm)	Fe(II)-O_2_ (nm)
***Mtb***					
DosS full	406, 500, 630 ^a^	428, 562 ^a^	422, 540, 570 ^a^	419, 547, 577 ^a^	414, 543, 578 ^a^
DosS GAFA	406, 550, 630 ^b^	428, 562 ^b^	422, 540, 570 ^b^	419, 545, 574 ^b^	540, 580 ^c^
DosT full	412, 528, 560 ^d^	430, 554 ^e^	422, 542, 568 ^e^	420, 546, 576 ^e^	415, 542, 578 ^e^
DosT GAFA	407, 539, 581 ^f^	432, 560 ^f^	421, 538, 568 ^f^	419, 549, 569 ^f^	414, 542, 577 ^f^
***Smeg***					
DosS full	407 ^g^	425, 500–600 ^g^		425 ^g^	413,576, 541 ^g^

^a^ [31], ^b^ [30], ^c^ [35], ^d^ [34], ^e^ [33], ^f^ unpublished, Ioanoviciu, A. ^g^ [25].

The crystal structure of the *Mtb* DosT GAF-A domain has been determined at 2.3 Å resolution in both the deoxy- and oxygen-bound states (Figure 3) [32]. The ferric protein was actually utilized, but x-ray photoreduction generated the ferrous protein *in situ*, part of which bound molecular oxygen. The distal and edge residues contacting the heme were Tyr98, Arg106, Ile109, Gly110, Ser111, Leu112, Glu114, Gly115, Arg116, Gly117, and Val118, all of which are within 4 Å of the heme. In one of the two monomers in the asymmetric unit, a water molecule was near the heme iron atom and hydrogen-bonded to the Tyr169 oxygen atom. This water is presumably that coordinated to the iron in the ferric state prior to x-ray photoreduction. In the second monomer, a molecule of oxygen was found stably coordinated to the heme iron atom with strong H-bonding to Tyr169. The iron-oxygen distance in the structure was 2.60 Å and the distance to the Tyr169 OH group was 2.50 Å. The tilt angle of the oxygen molecule relative to the heme plane was ~123° in the direction of the γ-*meso*-carbon [32]. The resonance Raman spectra of ^16^O_2_ and ^18^O_2_ bound to DosS support the presence of a strong hydrogen bond to the distal oxygen of the dioxygen ligand, an inference strengthened by the observation of changes in the spectra in H_2_O *versus* D_2_O [31]. 

The crystal structure of the *Mtb* DosS GAF-A heme domain in the ferric state revealed that a water ligand was coordinated to iron on the distal side (Figure 2) [35]. As found for DosT, the water ligand was lost on reduction of the DosS GAF-A domain, resulting in formation of a five-coordinated species. 

A hydrogen-bonding network in ferric DosS extending from the iron-coordinated water molecule through Tyr171, Glu87, and His89 [35] is also found in DosT, except that the residue that corresponds to Glu87 is replaced by a glycine (Gly85) [32]. In marked contrast to DosT, exposure of the reduced DosS protein to oxygen was reported to immediately produce the ferric protein via autooxidation rather than the ferrous dioxygen complex [35]. The authors subsequently proposed that steric constriction due to Glu87 in DosS makes it sensitive to autooxidation on oxygen binding, whereas the more open channel in DosT due to the Gly87 substitution enables stable oxygen binding [36]. To test this proposal, the DosS E87A and E87G mutants and, conversely, the DosT G85E mutant were prepared and their crystal structures determined. The authors report that the DosS E87A mutant now binds oxygen, whereas the DosT G85E mutant becomes autooxidizable. However, no actual autooxidation kinetics were reported and the interpretation of these otherwise interesting results is obscured by the discrepancy in the autooxidizability of DosS observed by different investigators.

Crystallographic and other evidence suggests that the hydroxyls of Tyr169 in DosT and Tyr171 in DosS are hydrogen bonded to molecular oxygen when it is bound to the heme iron atom. Tyr171 was therefore mutated to a phenylalanine to test the role of this residue in sensor function [37]. Strikingly, the Y171F mutant no longer discriminated between the O_2_, CO, and NO ligands, exhibiting no autophosphorylation activity in the presence of any of them. This loss of phosphorylation activity did not reflect gross disruption of the communication between the heme and kinase domains, as the ferrous deoxy Y171F mutant, in which no gaseous ligand is bound to the iron, retained kinase activity comparable to that of same state of the wild-type protein. Furthermore, the Y171F mutant of full-length DosS autooxidizes at essentially the same rate as the wild-type protein [38]. Nevertheless, UV-visible and resonance Raman spectroscopic analyses showed that the environment of the oxygen iron ligand is altered in the mutant from that of the wild-type, as expected if an H-bond between the tyrosine and the oxygen ligand is disrupted [13]. The Y171F mutation in DosS changes the K_d_ for CO from 8.29 to 3.33 μM, and that for oxygen from 0.58 to 0.12 μM [38]. Thus, replacement of the tyrosine by a non-hydrogen bonding phenylalanine, which suppresses the gas-specific response, results in tighter binding of both O_2_ and CO. The results suggest that interaction of the oxygen ligand with Tyr171 is important for ligand discrimination, but not for the stability of the oxygen complex or the ability to perform the autophosphorylation reaction.

The structures of the DosS and DosT GAF-A ferrous-CO and ferrous-NO complexes are not available, but a structure of the ferric-CN complex of the DosS GAF-A domain, which provides little additional insight, has been reported [35]. 

## 4. GAF-B Domain

The crystal coordinates of the *M. smegmatis* GAF-B domain (PDB 2VJW and 2VKS) have been reported, but so far do not clarify the role of GAF-B in the full-length protein [25]. The crystal structure coordinates of the GAF-B domain of DosT have also been deposited in the PDB (3ZXQ), but a paper reporting the work has yet to be published. 

The GAF-B domain of *M. smegmatis* DosS was shown to not bind cGMP or cAMP, in accord with similar findings for the *Mtb* GAF-B domain [28], and caused no increase in autophosphorylation rates. 

## 5. Kinase Domain

The crystal structures of the kinase cores of both DosS (residues 454–578, PDB 3ZXO) and DosT (451-residues 573, PDB 3ZXQ) provide information on the structure of the kinase domain and identify residues that are important for ATP binding, phosphorylation and communication with the N-terminal signaling core of the protein [39]. The kinase domains of the DosS and DosT proteins, which are structurally very similar, contain an ATP binding domain (ABD) and dimerization and histidine phosphate accepting domains (DHp). 

Crystal structures of other known histidine kinases have conserved N, F and G box elements essential for ATP binding and a long characteristic loop called the ATP lid motif in their ABDs that covers the nucleotide and protects it from unwanted reactions [40,41]. Interestingly, these two features are not present in the structures of the DevS and DosT kinase cores.

The crystal structure of the kinase domain of DosS determined at 1.8 Å resolution revealed the presence of two identical ABDs, each of them containing 3 α helices and 5 stranded β-sheets that together form a sandwich fold [37]. A short loop region connecting the α3 and β3 helices that is stabilized by strong hydrogen-bonding interactions and a zinc atom was found to be above the putative ATP binding pocket. This loop region substitutes for the ATP-lid motif found in other histidine kinases. Overall, the structure showed that the native state of the kinase domain’s active site is a closed conformation with the ATP binding site covered by the short loop. The kinase domain of DosT determined at 1.9 Å resolution revealed similar structural features with a closed conformation in the native state.

## 6. Domain Interactions

An early attempt to express full-length DosS and DosT yielded the proteins as inclusion bodies that, after solubilization, purification, and refolding, afforded proteins with autophosphorylation activity. It is virtually certain that these proteins lacked the heme prosthetic group due to the solubilization and refolding steps [13], which implies that the apo-protein has the ability to phosphorylate itself and to transfer the phosphate to DosR. In agreement with this, the DosS histidine kinase domain, DosS_201_, which lacks the GAF-A and GAF-B domains, was found to have autophosphorylation activity and site-specific mutagenesis identified His395 as the DosS autophosphorylation site [12,29]. These results indicate that the kinase domain is intrinsically catalytically active, and thus that the response to gases involves interactions with the rest of the full-length DosS protein that, for O_2_, results in inhibition of this activity. It is also relevant that the *M. smegmatis* H150A DosS protein, which binds no heme, lacked autokinase activity, but a construct consisting of the GAF-B and kinase domains was active under all conditions—*i.e*., the protein without the heme domain was active but no longer responsive to gas binding [25].

Resonance Raman comparison of full-length DosS and its truncated GAF-A domain indicates that two conformers exist of the Fe-CO complex in the truncated protein, whereas only a single conformer is detected for the full-length protein. This finding indicates that interactions between GAF-A and the other two domains in full-length DosS alter the GAF-A heme environment [15,38]. A more extensive comparison of GAF-A, a GAF-A/GAF-B construct, and the full-length protein in the ferric and ferrous states with CO, NO, or O_2_ bound, confirms this inference [31]. Fusion of the GAF-B to the GAF-A domain increases the specificity of the binding of CO and NO *versus* O_2_ to the heme. Thus, whereas two populations of CO and NO complexes are observed with the isolated GAF-A domain, only one conformer is significantly present in the GAF-A/GAF-B or full-length proteins. The binding of O_2_, however, is essentially the same in all the constructs and is consistent with preservation of a hydrogen-bonded network that stabilizes the O_2_ in a specific conformation [31].

The rate of autooxidation of the DosS ferrous dioxygen complex decreases in going from the isolated GAF-A domain, to the GAF-A/GAF-B fusion, and finally to the full-length protein, with t_1/2_ values of 16.5, 26.0, and 73.6 h, respectively [38]. Interaction of the GAF-A heme domain with the other domains thus stabilizes the ferrous-dioxygen complex with respect to autooxidation. 

Measurements of the CO association and dissociation constants for the same three protein constructs indicates that, although the differences are small, the association constants *k_on_* increase in the order GAFA (0.18 ± 0.007 μM^−1^·s^−1^) < GAF-A/GAF-B (0.26 ± 0.024 μM^−1^·s^−1^) < full-length DosS (0.31 ± 0.031 μM^−1^·s^−1^) [38]. In contrast, the *k_off_* values are virtually unchanged (2.57–2.74 s^−1^) for the three proteins (Table 3). 

**Table 3:** Equilibrium and kinetic values for DosS and DosT.

		O_2_			CO		NO
Protein	*k_on_*	*k_off_*	K_d_	*k_on_*	*k_off_*	K_d_	K_d_
	M^−1^·s^−1^	s^−1^	M	M^−1^·s^−1^	s^−1^	M	M
DosT	0.79 ^a^	20 ^a^	26 ^a^	0.05 ^a^	0.06 ^a^	0.94 ^a^	0.005 ^a^
DosS	8.8 ^a^	12.5 ^a^	3.0 ^a^	1.8 ^a^	0.06 ^a^	0.036 ^a^	0.020 ^a^
DosS	11.8 ^b^	6.79 ^b^	0.58 ^b^	0.31 ^c^	2.57 ^c^	8.29 ^c^	

^a^ Values in Tris^.^HCl buffer, 50 mM KCl, 5% ethylene glycol, at pH 8.0, 25 °C [33]; ^b^ Values in phosphate buffer, 200 mM NaCl, 1 mM EDTA, pH 7.5, 25 °C [38]; ^c^ Values in phosphate buffer, 200 mM NaCl, 1 mM EDTA, pH 7.5, 4 °C [38].

## 7. Heme Iron Reduction

*In vitro*, the endogenous *Mtb* ferredoxin reductase/ferredoxin pair encoded by the genes Rv0688 and Rv0763c rapidly reduces full-length ferric DosS (*k* = 0.0027 s^−1^, t_1/2_ = 4.4 min) to the ferrous state [38]. However, it should be noted that ferredoxin fdxA (Rv2007c) is part of the DosR regulon and is one of its most highly induced genes. It was reported to be induced 22–26-fold under hypoxia [26,42] and an independent comparison of its induction by ascorbate under aerobic conditions in *ΔdosT* *versus* *ΔdosS* knockout strains indicated that *fdxA* was induced 82-fold in a *dosS*-specific manner [43]. FdxA may therefore play an even more significant role in the physiological reduction of DosS and DosT. 

As mentioned, the *dosR* regulon is induced by ascorbic acid under both hypoxic and normoxic conditions [43,44]. One of these studies attributed the induction by ascorbate to oxygen depletion [44], but the other, carried out at shorter time periods after ascorbate addition, did not find a correlation between induction and increasing anaerobicity [43]. In this latter study under normoxic conditions, induction of the DosR regulon by ascorbic acid was impaired by chlorpromazine, CSU-20, and nitrate, agents that either decrease electron flow into the menaquinone pool or decrease the size of that pool [43]. A smaller reduction in signaling was caused by these agents under anaerobic conditions. Conversely, exogenous addition of vitamin K2, a menaquinone analogue, enhanced DosR regulon expression. These results suggest that electron delivery to the DosS signaling system by the mycobacterial central electron transport system is important for DosS/DosR signaling. Nevertheless, the interpretation of these results is unclear. Reduction of DosS under aerobic conditions should lead to formation of the non-phosphorylating ferrous dioxygen complex, and thus should not result in induction of the DosR regulon. If DosS autooxidation is nearly instantaneous, as reported by some investigators, DosS would be mostly in the ferric state, again a non-phosphorylating form of the protein. In the absence of CO or NO, the only state that would lead to an increased expression of the DosR regulon would be the ferrous deoxy form of DosS, which would suggest that DosS is largely in the ferric state in intact *Mtb* and must be reduced to the ferrous state before it can respond to gaseous stimuli. 

Non-enzymatic agents have been used to directly reduce DosS *in vitro*. Ascorbate can reduce DosS *in vitro*, although ascorbate reduces the DosS ferric-NO complex to the ferrous-NO complex more rapidly than it does the unliganded ferric protein [45]. As noted above, ascorbate induces the DosR regulon in intact mycobacteria under aerobic conditions in a *dosS*-dependent manner, but ascorbate probably acts in this instance by reducing cytochrome c and thus increasing the availability of reducing equivalents [43]. *In vitro*, flavin nucleotides in the presence of dithionite accelerate the reduction of ferric DosS compared to the reduction mediated by dithionite alone, but NADH does not directly reduce the protein [35].

## 8. Ligand Recognition and Communication

**Figure 4:**
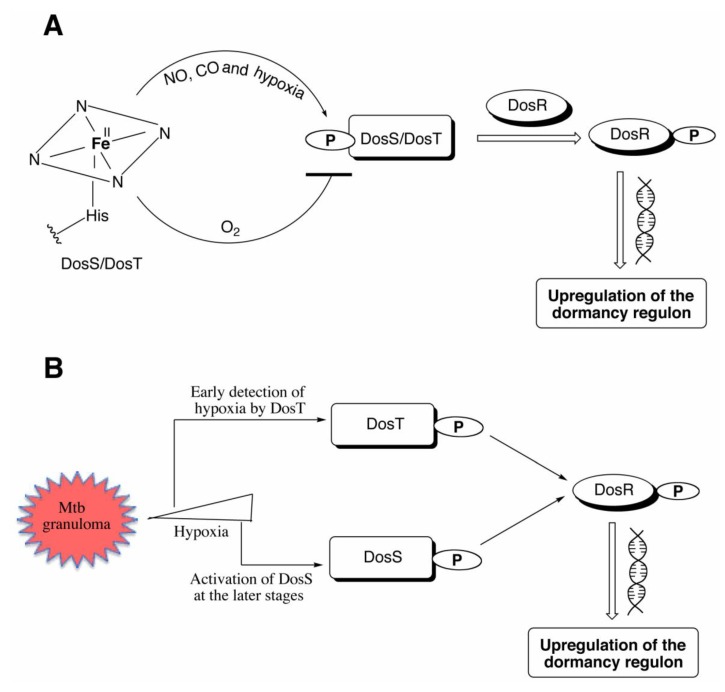
(**A**) The deoxy ferrous form of DosS/DosT is autophosphorylated under hypoxia or upon binding CO or NO, while no phosphorylation is observed when the proteins bind oxygen. The phosphorylated protein transfers the phosphate group to DosR, which then binds DNA resulting in downstream signaling leading to the upregulation of the 47 other genes necessary for dormancy. (**B**) DosT is sensitive to low oxygen concentrations inside the cell and responds during the early transition from aerobic to hypoxia by autophosphorylation and induction of the *dosR* regulon. DosS detects hypoxia at later stages and is responsible for maintaining the dormancy regulon induction.

Figure 4(A) depicts a general scheme indicating the ligands that trigger and suppress the autophosphorylation in DosS and DosT, leading to downstream signaling and upregulation of the dormancy regulon. In spite of our wide understanding of the nature of the ligands that activate/deactivate the signaling in these proteins, the actual biochemical mechanisms of ligand recognition and communication remain obscure. Tyr171 is responsible for ligand discrimination as its mutation to a Phe renders the protein inactive in all ligand bound states [37], although it retains normal activity in the ligand-free deoxy-ferrous state, which indicates that the mutation has not disabled the ability of the protein to autophosphorylate. Interaction of the tyrosine with the distal ligand thus appears to be essential for ligand discrimination and ligand-dependent activity. The OFF state is defined by a strong hydrogen-bonding interaction between the tyrosine hydroxyl group with the distal oxygen of the iron-bound ligand, which may lock the protein active site in a particular conformation. A comparable interaction can be envisioned for the met (Fe(III)) form of the protein, which is also inactive, in that the distal iron-bound water molecule, as shown by resonance Raman and the crystal structure, forms a hydrogen bond with the tyrosine.

In this view, the ON state of the protein is characterized by the absence of a strong hydrogen bonding interaction of the distal ligand with Tyr171. This may allow the tyrosine to partner with other hydrogen bond donors in the distal pocket, which directly or through a conformational change may be responsible for communication with the phosphorylation domain. For example, the tyrosine was shown in the crystal structure of the DosS GAFA domain to be hydrogen-bonded to Glu87, which in turn is hydrogen-bonded to His89 (Figure 2). Alternatively, the minor conformer in which the tyrosine is hydrogen-bonded to the distal ligand (CO or NO) was also postulated to be responsible for the ON state. However, the overall role of the distal hydrogen-bonding chain in sensing and signaling remains unclear. The distal E87G and E87A mutants were reported recently to form stable ferrous-oxy complexes, but no information is available on their kinase activity, as they were only examined in the truncated GAF-A domain and not in the full-length protein [36].

The activity of the ligand-free Fe(II)-deoxy state may arise from the release of steric or hydrogen-bonding constraints in the distal pocket. The hydrogen-bonding interactions of the tyrosine to other distal residues, as seen in the CO and NO bound forms, are clearly not critical for activity, as the Y171F mutant that has no hydroxyl group retains its activity in the deoxy form. It is likely that in the deoxy state, the protein is able to sample both active and inactive conformations, giving rise to an autophosphorylation activity that is intermediate between the high activities of the CO or NO-bound states and that of the inactive O_2_-bound state.

Recent crystal structures of the DosS and DosT kinase domains [39] reveal a closed conformation of the active ATP binding site that required a major conformational change to bind ATP. Interaction between the HisKA domain, containing the phosphate accepting histidine, and the ATP binding domain (ABD) activates the ATP binding by displacing the short loop that covers the ATP binding pocket and allows the nucleotide to bind and then to phosphorylate the histidine. Specifically, ionic interactions between residues R440 and E537 in the histidine kinase and ABD domains, respectively, were shown to be responsible for effecting the autophosphorylation. Accordingly, mutations of these residues to cysteines resulted in reduced levels of autophosphorylation activity.

On the response regulator side, the Asp54 that is phosphorylated in DosR to initiate the signaling cascade was shown to be involved in a protein-protein interaction with the DosS to complete the phosphotransfer reaction. Accordingly, a D54A mutant suppressed interaction of the DosR and DosS proteins, as revealed by a pull down assay. Furthermore, a conserved residue, Lys104, is expected to sense the phosphorylation of DosR and the conformational changes associated with it by forming a hydrogen bond with the phosphorylated Asp54. Mutation of Lys104 to alanine results in no phosphorylation of DosR by DosS and renders it functionally inactive *in vivo*, although it doesn’t seem to perturb the protein-protein interaction between DosR and DosS [46].

## 9. Roles of DosS and DosT

The relative roles of DosS and DosT in inducing the *Mtb dosR* regulon were examined by comparative microarray analysis of Δ*dosS*, Δ*dosT*, and the double mutant in the Wayne anaerobic model [16]. The results indicated that DosT, which is not part of the DosR regulon, initiates the response to hypoxia or NO and induces the *dosR* regulon. DosS is part of that regulon, so it is itself induced, eventually to a level as much as 100-fold greater than that of DosT. DosS then becomes the primary receptor that maintains the induction of the *dosR* regulon. Both DosT and DosS are required for full induction and survival of *Mtb* under hypoxia [16]. A *dosR* knockout showed that DosR is required for survival of *Mtb* under hypoxic conditions and for its transition from hypoxic to normoxic metabolism [47].

As already mentioned, although* M. smegmatis* has a DosS but not a DosT sensor, its DosS is closer in sequence to *Mtb* DosT than DosS [24,25]. Furthermore, like *Mtb* DosT, the *smegmatis* DosS is purified in the ferrous state that binds oxygen and is resistant to autooxidation [25]. In a follow-up study, *MtbdosT*, but not *dosS*, was found to substitute functionally for *dosS* in an *M. smegmatisdosS* knockout [24]. The *dosR* regulon is induced mostly during the early phase of the transition from aerobic to hypoxic conditions, consistent with the fact that DosT responds to a decrease in oxygen tension more sensitively and strongly than DosS (Figure 4(B)). Complementation of an *M. smegmatis* DosS knockout with chimeras in which the GAF-A domains were exchanged between DosS and DosT showed that the difference in the ability of DosS and DosT to substitute for DosS in *M. smegmatis* resides in the GAF-A domains. Sequence alignments showed that 7 residues are conserved differentially across species between the *Mtb* DosS and DosT GAF-A domains. Replacement of the individual amino acids of DosS by those of DosT showed that the E87G, D90G, H97E, L118R, and T169N mutants functioned in *M. smegmatis*, whereas the H89R and V108R forms did not [24]. Thus, just one substitution of DosS Glu87, Asp90, His97, Leu118, or Tyr169 with the corresponding DosT residue was sufficient to enable the *Mtb* DosS mutant to function like DosT in *M. smegmatis*. This led the authors to propose that DosS and DosT are not redundant, but rather play different roles [24]. 

Microarray analyses of gene expressions in wild-type *Mtb* and a *dosR* knockout strain indicated that fifty-two genes are significantly upregulated, while 19 genes are downregulated by hypoxia [48]. *DosR* regulon expression was increased by hypoxia, exposure to nitrosoglutathione, ethanol, and to a lesser extent, H_2_O_2_, but not after heat- or cold-shock. The *dosR* knockout was also used to demonstrate that the *ΔdosR* strain was more virulent, with significantly shorter survival times when it was used to infect immunocompetent mice instead of the wild-type strain [49]. 

A comparison of the abilities of *ΔdosS* and *ΔdosT Mtb* knockouts to induce two representative proteins of the *dosR* regulon showed that changes in the electron transport system, such as those caused by ascorbate under aerobic conditions, induce the *dosR* regulon in the *dosT* but not *dosS* knockouts [43]. Furthermore, the *fdxA* gene, which codes for a ferredoxin, is induced in the *ΔdosT* but not *ΔdosS* strain. Addition of chlorpromazine, a type II oxidoreductase inhibitor, and CSU-20, a menaquinone synthesis inhibitor, to hypoxic cultures markedly decreased signaling, but no effect was seen with aerobic cultures unless nitrate, an alternative electron acceptor, was also added. Thus a decrease in electron transport correlates with decreased signaling, and an increase with increased signaling. Addition of vitamin K1 or K2 gave higher induction of the *dosR* regulon [43]. The authors concluded that DosS senses the redox status of the cell and argue that DosS is both an oxygen sensor (off switch) and redox sensor (on switch). Increasing electron donation ability, as suggested, should enhance reduction of the ferric to the ferrous state of the sensors. 

Heme oxygenase-derived CO induces the *dosR* regulon [15] and some evidence has been obtained that CO is primarily sensed via DosS, with DosT playing a lesser role. This would appear to contradict the hypothesis that DosS is a redox as opposed to a gas sensor. Recent evidence has shown that heme is degraded in *Mtb* by an oxygen-dependent enzyme, MhuD, via an unusual mechanism that does not produce CO [50]. All other known heme oxygenases cleave the heme ring with the production of a molecule of CO. Heme degradation without the formation of CO would allow the mycobacteria to scavenge the heme iron atom without triggering the dormancy regulon via DosS/DosT sensing.

Microarray expression profiling has shown that low concentrations of cyanide suppress induction of the *dosR* regulon by NO or hypoxia, and does so without significantly influencing expression of genes in the regulon. The effect of cyanide was specific, as it did not modulate overall gene transcription or induction even at 10-fold higher concentrations than those required to block NO or oxygen signaling [2]. As cyanide only binds to ferric DosS and DosT, this result could be interpreted to mean that cyanide acts by locking one or both of these sensors in the ferric (inactive) state. Another study in which the expression of the reporter protein *hspX* was highly suppressed in an aerobically grown *M. smegmatis* culture with added KCN implies that CN^−^ binds to the ferric heme and that the heme in the native DosS/DosT proteins is in the ferric state [25]. 

## 10. DosS—Gas or Redox Sensor?

The ferrous dioxy form of DosT has been found to be stable in the hands of all the investigators who have purified and characterized this protein. Its rate of autooxidation is very low (< 0.01 h^−1^) [33]. It is therefore generally agreed that DosT is a gas sensor with little kinase activity in the ferric state and a moderate kinase activity in the ligand-free ferrous state that is enhanced by the binding of NO or CO and suppressed by the binding of O_2_. 

In contrast, there is disagreement about the stability of the ferrous dioxygen form of DosS, and consequently about whether it functions as a gas or redox sensor. Steyn and coworkers reported that DosS was purified from their expression system as the ferric protein. This protein could be reduced anaerobically to the ferrous state, but on exposure to oxygen reverted to the ferric state within seconds [34]. Formation of the ferric protein on oxygen exposure was supported by the EPR spectrum of the final protein, which had a signal at g = 5.98 characteristic of a ferric high-spin iron. Furthermore, spectroscopic evidence showed the protein bound cyanide and was not altered by exposure to ferricyanide. These data are consistent with formation of the ferric protein. 

Cho *et al*., in preparing the DosS GAF-A domain for crystallographic studies, similarly found that in their hands exposure of the ferrous protein to oxygen yielded the ferric protein with little evidence for the ferrous dioxy complex [35]. Restricted access in the channel leading to the heme iron was suggested to be responsible for the instability of the oxy-complex. A network of hydrogen bonding interactions involving water molecules and the propionate side-chain was proposed to mediate electron transfer between oxygen and the ferrous heme iron, making it a redox sensor. Glu87 in the channel was proposed to be the residue hindering access of the oxygen to the heme, a hypothesis that was supported by the finding that mutation of Glu87 to a Gly or Ala gave a protein with a stable oxy-ferrous complex [36]. Conversely, mutation of the native Gly85 in DosT, which does form a stable oxy complex, to a Glu resulted in oxidation of the iron. It is not clear, however, why restricted access caused by Glu87 would apply only to oxygen, as CO and NO bind normally and, in any case, why restricted access would alter the stability of the oxygen complex. 

In contrast, the rates of autooxidation of the ferrous-dioxygen complexes of the DosS GAF-A, GAF-A/GAF-B, and full-length proteins in various buffers in the presence of various specific cations have been exhaustively measured by UV-visible spectroscopy [38]. The intrinsic autooxidation rates of all three protein constructs were very low at 25 °C and pH 7.5, with autooxidation rates in the order of 0.003–0.005 h^−1^ and half-lives greater than 24 h in the presence of K^+^, Na^+^, Mg^2+^, or Ca^2+^ (Table 4). Only high concentrations of copper ions greatly decreased these half-lives. Surprisingly, mutation of Tyr171, the distal tyrosine, to a phenylalanine in the full-length DosS protein did not increase the autooxidation rate [38]. 

Slow autooxidation rates were also observed for the full-length proteins by Sousa *et al*. [33], who in addition to the autooxidation rates also measured the k_on_, k_off_, and K_d_ values for the O_2_, NO, and CO complexes (Table 3, Table 4). They found t_1/2_ = 4 h for autooxidation of full-length DosS at 37 °C and pH 8.0. 

**Table 4:** Aerobic K_ox_ values for DosS.

Hepes 20 mM, pH 7.5 (25 °C) ^a^	*k* (h^−1^)	*t*_1/2_ (h)	Reference
no additions	0.003	210	[38]
+ 200 mM KCl	0.013	78	[38]
+ 200 mM NaCl	0.010	96	[38]
+ 0.1 mM CaCl_2_	0.005	170	[38]
+ 1.0 mM MgCl_2_	0.012	59	[38]
+ 0.1 mM CuCl_2_^b^	25	0.03	[38]
+ 10 μM CuCl_2_^b^	0.062	11	[38]
+ 0.1 mM FeCl_3_^b^	0.089	7.8	[38]
phosphate EDTA^c^	0.019	36	[38]
Tris 50 mM, 50 mM KCl, 5% ethylene glycolpH 8.0 (37 °C)	0.17	4	[33]

^a^ Hepes pretreated with Chelex 100 resin. ^b^ The cations indicated were added as salts of high purity (>99.999%). ^c^ In phosphate buffer (50 mM, pH 7.5), 1 mM EDTA, and 200 mM NaCl. ^d^ The nominal FeCl_3_ concentration is indicated.

The ferrous dioxygen complexes of DosS and DosT have little kinase activity, but autokinase activity is observed with the ferrous-NO complexes. Stopped flow UV-vis and freeze-quench resonance Raman have been used to show that the ferrous dioxygen complex reacts with NO in a reaction that oxidizes the heme group to the ferric state with concomitant production of nitrate (NO_3_^−^) [45]. The reaction is biphasic with k_obs_ = 36 and 1.2 s^−1^ at pH 7.5 with 2 μM NO. Ferric DosS does not have autokinase activity but binds NO with high affinity (K_d_ ~ 5 μM) to give the ferric-NO complex that is readily reduced by ascorbate to the active ferrous-NO complex. Thus, exposure to low levels of NO can convert the inactive DosS ferrous-oxygen complex to the active ferrous-NO complex by a sequence involving oxidation followed by reduction in the reducing environment of *Mtb* cells [45]. 

Ultrafast time-resolved absorption spectroscopic analysis of the dissociation of O_2_, CO, and NO from the truncated GAF-A domains of DosS and DosT indicates that the heme pocket in both proteins is relatively closed [51]. Upon photodissociation, only 1.5% of the O_2_ escapes from the DosT heme pocket on the picosecond time scale. This value, which is similar to that for other hemoprotein oxygen sensors, shows that it is an effective oxygen trap. In contrast, 18% of the oxygen escapes under similar conditions from the DosS GAF-A domain. The heme environment in DosT thus prevents oxygen escape to a much greater extent than that of DosS. Distal Tyr169 in DosT plays a role in constraining the oxygen, as its mutation to a phenylalanine results in a much higher (19%) oxygen escape. In contrast, the fraction of CO that rebinds in the heme pocket is higher for DosS than for DosT. NO rebinding is similar for both proteins, but is substantially slower for the ferric than ferrous states. These differential effects involved the distal tyrosine, but not exclusively, as mutation of other nearby residues in some instances also affected dissociation and rebinding of ligands. The data are consistent with a weaker hydrogen-bond between Tyr171 and the dioxygen ligand in DosS than that from Tyr169 in DosT [51]. The results do not, however, provide support for a critical steric effect on oxygen binding and dissociation and the autooxidizability of DosS. 

Finally, the purified DosS from *Mycobacterium smegmatis* displayed significant autokinase activity in presence of the ubiquinone/ubiquinol pool, indicating that the redox state of the quinone/quinol did not affect the kinase activity [25]. This was further supported by *in vivo* studies in which mutations to cytochrome bc1-aa3, a critical component in an electron transport pathway involving the menaquinone pool, led to inhibition of the growth of the *M. smegmatis* cultured under aerobic conditions, but failed to induce the DosR regulon [52]. These results are inconsistent with a role for DosS as a sensor of the redox state of the cell. 

## 11. DosR

Phosphorylation of DosR by DosS is required for activation of the regulon. The phosphorylation involves formation of a complex of DosS with DosR, as shown by experiments in which subunits of dihydrofolate reductase were attached to these two proteins and reconstitution of dihydrofolate reductase activity was used as a marker of complex formation [53].

A study of the regulation of *dosR*/*dosS* in *M. smegmatis* under aerobic conditions showed that the DosR protein binds to a conserved DNA motif located upstream of the* acr* gene of *Mtb* [54]. Evidence that phosphorylation of DosR is required for turning on the *dosRS* operon, the sites of binding of DosR to DNA, and the need for recruitment of a second molecule of phosphorylated DosR for gene activation has been reported [55]. Phosphorylated DosR binds to a Dos box-like sequence on DNA to upregulate transcription of narK2 and Rv1738, which codes for a nitrite-nitrate transporter, presumably because nitrate influx is used to maintain the redox balance or to provide energy during the shift-down of metabolism to dormancy [56].

DosR, a member of the NarL subfamily of response regulators, has an N-terminal domain that includes Asp54, the phosphorylation site, linked via a connecting sequence to a C-terminal DNA-binding domain. Asp54 was identified as the DosR phosphorylation site by mutagenesis [12]. Mutagenesis has also shown that Asp8 and Asp9 are important for transfer of the phosphate from DosS to DosR, as mutation of these residues prevents the transfer. The two residues are postulated to be involved in binding a catalytically important Mg^2+^ ion [12]. The equivalent of Thr82 in other NarL regulators is critical for their function, but the crystal structures show that Thr82 in DosR is relatively distant from other critical residues. Nevertheless, the T82A mutant transfected into a Δ*dosR* *Mtb* strain fails to induce the *dosR* regulon. Thr82 thus appears to be essential despite is somewhat anomalous topological position [57].

A comparison of the truncated C-terminal DNA-binding domain of DosR with the binding of the full-length protein shows that the isolated C-terminal domain can bind to DNA at the same high affinity target promoter site [58]. One role of the N-terminal domain, which includes the phosphorylated residue, is therefore to mask the intrinsic DNA affinity of the C-terminal domain. Furthermore, unlike phosphorylated full-length DosR, the C-terminal domain does not interact with a vicinal low affinity site. This suggests that the N-terminal domain is also involved in promoting cooperative binding at this second site. As a result, the C-terminal domain alone weakly induces the DosR regulon under aerobic conditions, but it is unable to support gene activation during hypoxia [58]. 

The crystal structures of the full-length, unphosphorylated *Mtb* DosR and of its C-terminal domain at 2.2 Å and 1.7 Å resolution, respectively, have suggested that a helix rearrangement following phosphorylation may be responsible for DosR activation [59]. Analysis of the structure of the C-terminal domain and of its complex, with a consensus DNA sequence of the hypoxia-induced gene promoter, shows that it contains four α-helices [60]. The C-terminal domain forms two dimers, which then assemble into a tetramer. On complexation with DNA, each DosR C-terminal domain inserts its DNA-binding helix into the major groove, causing two bends in the DNA. Three amino acid residues in each subunit, Lys179, Lys182, and Asn183, make multiple protein-DNA base contacts [60].

## 12. Inhibition of DosS-DosT/DosR

In principle, inhibition of the DosS-DosT/DosR sensor system would hinder entrance into the latent state and might result in reversion from the latent to the active state. Given the much higher sensitivity of the active form of *Mtb* to drugs, forcing it to remain in the active state may constitute an approach for more effective suppression of the persistent, latent state of the mycobacteria. 

**Figure 5:**
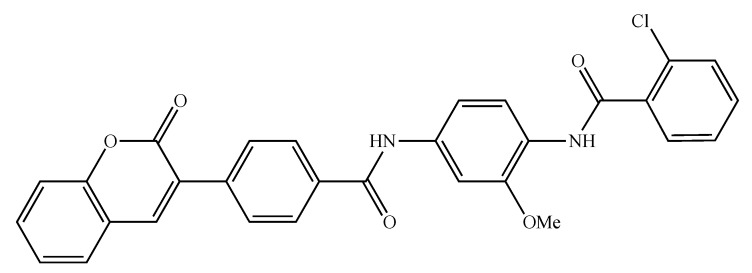
Structure of DosR inhibitor [62].

Although little work has yet been done in this direction, a homology-based model of DosR was used to identify a phenylcoumarin derivative (Figure 5) that inhibits binding of DosR to the target DNA, downregulates dormancy gene transcription, and reduces survival of hypoxic dormant bacteria. The authors postulate that the compound “locks” DosR in an inactive conformation that does not bind to DNA [61,62]. A high-throughput microplate phosphorylation assay has also been implemented to screen for antituberculosis compounds acting as DosR inhibitors [61].

## 13. Conclusions

DosS and DosT are autophosphorylating heme-dependent sensors that, together with the DNA-binding protein DosR, control the expression of a family of genes that are essential for entry into and survival in the latent, dormant state. The DosS and DosT proteins, under conditions that cause their activation, autophosphorylate a conserved histidine residue and then transfer it to DosR. Phosphorylated DosR then binds to DNA regulatory elements to alter gene expression. Two major questions remain concerning the structures and mechanisms of DosS and DosR. First, although crystal structures exist of the isolated DosS/DosT GAF-A domains in the ferrous, ferric, and oxygen bound states, no structures are available of the NO or CO ligated forms. Subtle differences that may exist in the heme domain between the OFF O_2_-bound state and the ON NO- or CO-bound states therefore remain unknown. Furthermore, although crystal structures are also available of the truncated GAF-B and kinase domains of DosS, no structure exists of the entire protein. A consequence of these gaps in our knowledge is that the mechanism by which the protein discriminates between the ligands and transmits the binding of activating ligands to the kinase domain remains obscure. The second question derives from the contradictory findings on the stability of the ferrous DosS oxygen complex. Detailed studies of the DosS and DosT GAF-A domains have shown that the ferrous dioxygen complex is not only isolable, but in fact quite stable. The stabilities of the ferrous dioxygen complexes are even greater in the full-length protein, with half-lives in the order of tens of hours. However, in some laboratories, similar constructs have been reported to undergo almost instantaneous autooxidation of the ferrous to the ferric state on exposure to oxygen. Although detailed kinetics for these autooxidation reactions have not been reported, it is clear that critical differences exist in the proteins and the conditions used for their study in different laboratories. The ambiguities introduced by these differences have raised the possibility that DosS is primarily a redox state sensor, unlike DosT, which is generally agreed to be a gas sensor. It is clear that DosS, at least some of the time, acts as a gas sensor, as it functions normally in the ferrous state and when either CO or NO is the bound ligand. If DosS were a redox sensor, one would expect that the ability of the mycobacteria to reduce the ferric to the ferrous state would be a critical determinant of the response, a possibility that requires further exploration. However, in view of the demonstrated aerobic stability of the DosS oxygen complex in at least some hands, it is simplest to view both DosT and DosS as gas sensors until more conclusive evidence for an alternative role for DosS is forthcoming. 
